# Prediction of vitamin interacting residues in a vitamin binding protein using evolutionary information

**DOI:** 10.1186/1471-2105-14-44

**Published:** 2013-02-07

**Authors:** Bharat Panwar, Sudheer Gupta, Gajendra P S Raghava

**Affiliations:** 1 Bioinformatics Centre, Institute of Microbial Technology (CSIR), Sector 39A, Chandigarh, India

**Keywords:** Vitamin-interacting residue, Pyridoxal-5-phosphate, SVM, PSSM, VitaPred

## Abstract

**Background:**

The vitamins are important cofactors in various enzymatic-reactions. In past, many inhibitors have been designed against vitamin binding pockets in order to inhibit vitamin-protein interactions. Thus, it is important to identify vitamin interacting residues in a protein. It is possible to detect vitamin-binding pockets on a protein, if its tertiary structure is known. Unfortunately tertiary structures of limited proteins are available. Therefore, it is important to develop *in*-*silico* models for predicting vitamin interacting residues in protein from its primary structure.

**Results:**

In this study, first we compared protein-interacting residues of vitamins with other ligands using Two Sample Logo (TSL). It was observed that ATP, GTP, NAD, FAD and mannose preferred {G,R,K,S,H}, {G,K,T,S,D,N}, {T,G,Y}, {G,Y,W} and {Y,D,W,N,E} residues respectively, whereas vitamins preferred {Y,F,S,W,T,G,H} residues for the interaction with proteins. Furthermore, compositional information of preferred and non-preferred residues along with patterns-specificity was also observed within different vitamin-classes. Vitamins A, B and B6 preferred {F,I,W,Y,L,V}, {S,Y,G,T,H,W,N,E} and {S,T,G,H,Y,N} interacting residues respectively. It suggested that protein-binding patterns of vitamins are different from other ligands, and motivated us to develop separate predictor for vitamins and their sub-classes. The four different prediction modules, (i) vitamin interacting residues (VIRs), (ii) vitamin-A interacting residues (VAIRs), (iii) vitamin-B interacting residues (VBIRs) and (iv) pyridoxal-5-phosphate (vitamin B6) interacting residues (PLPIRs) have been developed. We applied various classifiers of SVM, BayesNet, NaiveBayes, ComplementNaiveBayes, NaiveBayesMultinomial, RandomForest and IBk etc., as machine learning techniques, using binary and Position-Specific Scoring Matrix (PSSM) features of protein sequences. Finally, we selected best performing SVM modules and obtained highest MCC of 0.53, 0.48, 0.61, 0.81 for VIRs, VAIRs, VBIRs, PLPIRs respectively, using PSSM-based evolutionary information. All the modules developed in this study have been trained and tested on non-redundant datasets and evaluated using five-fold cross-validation technique. The performances were also evaluated on the balanced and different independent datasets.

**Conclusions:**

This study demonstrates that it is possible to predict VIRs, VAIRs, VBIRs and PLPIRs from evolutionary information of protein sequence. In order to provide service to the scientific community, we have developed web-server and standalone software *VitaPred* (http://crdd.osdd.net/raghava/vitapred/).

## Background

A protein individually utilizes only a limited range of functionality present in its natural amino acid side chains, and the catalytic activity of many enzymes requires the involvement of a small-molecule that acts as a co-factor. These are required in almost all important metabolic pathways because they are specialized in certain types of reaction. One particular cofactor can be involved in several pathways and, conversely, several cofactors can be required in one particular pathway [1,2]. Many vitamins have diverse biochemical functions but they are primarily known to assist enzyme-substrate reactions by playing the role of an enzyme cofactor [3,4]. Some vitamins have hormone-like function as regulators of mineral metabolism (e.g. vitamin D), or regulators of cell and tissue growth and differentiation (e.g. some forms of vitamin A). The function of vitamin D as anti-infectious and anti-inflammatory is well-established [5,6] and other functions as antioxidants (e.g. vitamin E and sometimes vitamin C). The majority of vitamins (e.g. B complex vitamins) function as precursors of enzyme cofactor that helps enzyme in their work as catalysts in metabolism [7].

As most vitamin biosynthetic pathway enzymes are not present in mammals and present in many of the pathogens [8], these enzymes have become attractive drug targets in several disease including tuberculosis [8,9] and malaria [10,11]. Several investigators have targeted Ornithine decarboxylase (ODC) for different diseases like African trypanosomiasis, *Pneumocystis carinii* pneumonia, ischemia, autoimmune diseases and hyperplasia [12]. Nonetheless, many groups are targeting Serine hydroxyl-methyltransferase (SHMT) as antitumor target knowing that enhanced levels of SHMT activity have been found in rapidly proliferating tumor cells [13]. A constitutive ODC activity observed in cancer cells, where its uncontrolled expression confers a cancer phenotype to the cells so ODC has been targeted in antitumor drugs [14]. In past, several studies have been done to identify the cofactor binding cleft and interacting residues in various enzymes. Pyridoxal 5'-phosphate (PLP)-dependent enzymes like 3,4-dihydroxyphenylalanine decarboxylase (DDC) [15,16], Cystathionine beta-synthase (CBS) [17], 8-amino-7-oxononanoate synthase [18], Aminobutyrate aminotransferase [19], ODC and SHMT etc. have been investigated in various studies for identification of PLP and substrate interacting residues. These studies helped them to investigate the underlying mechanism and develop strategies for inhibitor designing. Similarly enzymes involved in folate (Vit-B9) metabolism such as Dihydropteroate synthase [20], Dihydrofolate synthase [21] and thiamin (Vit-B1) pathway [22] like Pyruvate dehydrogenase [23] and Oxoglutarate dehydrogenase [24] have also been taken as drug targets. In addition, binding of PLP also inhibits the activity of aminoacyl-tRNA synthetases [25]. Therefore, computational tool for the prediction of PLP and other vitamin-interacting site is highly desirable.

The advancement of genome sequencing produces huge amount of sequence data but reliable *insilico* annotation of these sequences still remains a challenge. There are several prediction tools available for the functional annotation of proteins. Broadly, the existing computational method can be divided in two categories; (i) protein level prediction, where function of whole protein is predicted [26-28] and (ii) residue level prediction where function of each residue in a protein is predicted [29-31]. The protein level prediction provides overall function of protein whereas residue level predictions are advancement over protein level and provides the information of functional residues. The residue level predictions mainly deal with prediction of interaction with other proteins, DNA, RNA and ligands. There are various methods to predict different interacting residues from the structure of protein but the major challenge is to predict interacting residues when only protein sequence is known. Several prediction methods have been developed for carbohydrates [32,33], lipids [34,35], DNA [29,36-39] and RNA [30,38,40] interacting residues in protein sequence. Some methods have been developed for specific ligands such as ATP [41,42], GTP [43], NAD [44], FAD [45] and mannose [46].

In this study, preliminary investigations revealed differential binding patterns of vitamins and other small-molecules. These differential patterns suggested that each ligand has specific residual preference for their binding with protein. Therefore, it becomes important to develop vitamin-specific interacting residue prediction methods. In this study, we developed different models for the sequence-based prediction of vitamin-interacting residues (VIRs), vitamin-A interacting residues (VAIRs), vitamin-B interacting residues (VBIRs) and PLP-interacting residues (PLPIRs). We utilized various classifiers and finally selected Support Vector Machines (SVMs) for developing the prediction models. SVM is a very powerful machine learning technique, which has been used for developing various bioinformatics methods in the past [38,47-50]. It has been shown that the evolutionary information provided more information [40,43,45] than protein sequence, therefore we applied evolutionary information in the form of Position-Specific Scoring Matrix (PSSM) profile for developing a prediction method. This vitamin binding site prediction will be very useful for the study of enzyme activity and further advancement of drug development technologies.

## Results

### Analysis of protein-binding patterns of various ligands

It is important to analyze protein-binding patterns of different ligands in order to understand binding specificity of each ligand. Previously published datasets of different ligand-binding patterns for example ATP, GTP, NAD, FAD and mannose, were used to look at the preference of interacting residues. We analyzed the ligand-binding patterns for ATP (Additional file 1: Figure S1), GTP (Additional file 1: Figure S2), NAD (Additional file 1: Figure S3), FAD (Additional file 1: Figure S4) and mannose (Additional file 1: Figure S5) with the help of Two Sample Logo (TSL) (See all Figures in Additional file 1). It was observed that each ligand preferentially interacted with different residues of proteins. The ATP, GTP, NAD, FAD and mannose preferred the residues {Gly, Arg, Lys, Ser, His}, {Gly, Lys, Thr, Ser, Asp, Asn}, {Thr, Gly, Tyr}, {Gly, Tyr, Trp} and {Tyr, Asp, Trp, Asn, Glu}, respectively. The non-preferred residues were {Leu, Ala, Pro, Glu, Val}, {Leu, Glu, Ile, Met, Val}, {Leu, Glu, Ala, Lys}, {Glu, Asp, Lys, Ala, Pro} and {Leu, Val, Ile} for the ATP, GTP, NAD, FAD and mannose ligands respectively. We further analyzed and observed that significant differences were also present in the neighboring residues surrounding these preferred and non-preferred sets. This suggests the existence of different binding pockets for each small molecule ligand in the proteins. In order to predict these potentially differing binding pockets, there should be ligand specific binding site tools.

### Analysis of different protein-interacting residues of different vitamin classes

**Figure 1:**
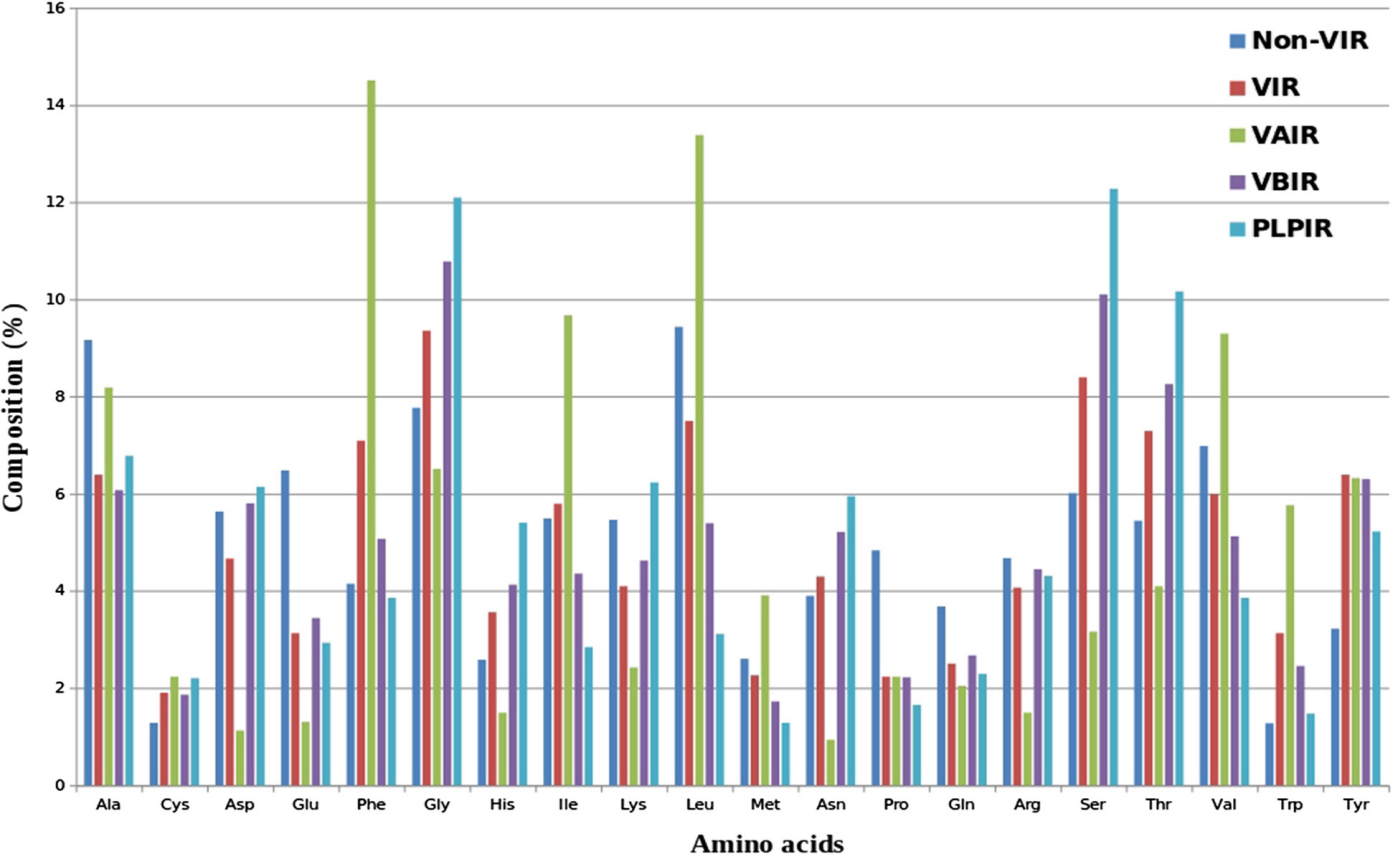
**Comparative average percent amino acids composition of VIRs**, **non**-**VIRs**, **VAIRs**, **VBIRs and PLPIRs.**

After analysis of various ligand-protein interactions, we compared vitamins-interacting patterns with other ligands and found that significant differences were present. The Tyr, Phe, Ser, Trp, Thr, Gly and His are preferred as VIRs whereas Glu, Ala, Pro, Leu, Lys, Gln, Val and Asp are non-preferred. We analyzed amino acid compositions of the vitamin binding protein residues grouped by the sub-class to which the binding protein belonged: VIRs, VAIRs, VBIRs and PLPIRs (Figure 1). The interacting site of Vitamin A, Vitamin B and PLP preferred {Phe, Ile, Trp, Tyr, Leu, Val}, {Ser, Tyr, Gly, Thr, His, Trp, Asn, Glu} and {Ser, Thr, Gly, His, Tyr, Asn} whereas the non-preferred residues were {Glu, Pro, Asp, Asn, Ser, Arg, Gln}, {Leu, Glu, Ala, Pro, Val, Ile, Lys} and {Leu, Glu, Ala, Pro, Val, Ile, Ala} respectively. This implies that differences do exist at the protein-vitamin interaction sites even within vitamins sub-classes.

In this study, we initially developed a model for the prediction of vitamin-interacting residues and then further classified VIRs into vitamin A, vitamin B and pyridoxal-5-phosphate (vitamin B6; PLP) interacting residues. Four different types of prediction methods were developed, one for each of the interacting residues: VIRs, VAIRs, VBIRs and PLPIRs. All the models developed in this study were evaluated using five-fold cross validation technique. In all cases, we used 10 times more negative instances than positive instances.

#### Prediction of vitamin-interacting residues (VIRs)

**Figure 2:**
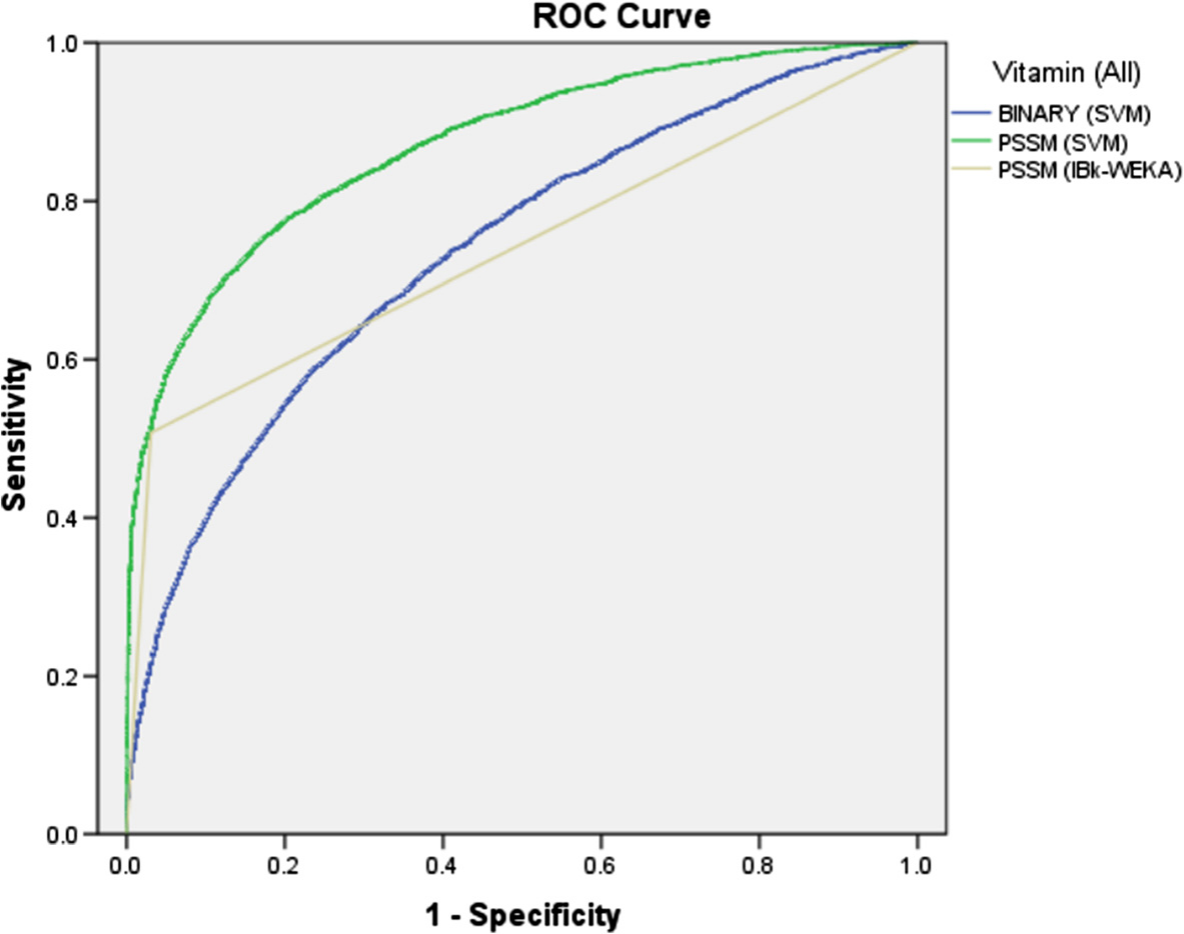
The ROC plot of the performance of different approaches for prediction of VIRs.

Here we developed the comprehensive prediction method for all VIRs. By generating sliding patterns and creating Two Sample Logo, we found that Phe, Gly, His, Ser, Thr, Trp and Tyr were more abundant in VIRs as compared to non-VIRs (See Additional file 1: Figure S6). These patterns were converted into binary patterns and different kernels/parameters of SVM were employed to optimize the discrimination power between VIR and non-VIR patterns. We achieved 68.57% sensitivity, 64.88% specificity, 65.22% accuracy and 0.20 MCC. Preferences for neighboring amino acids between VIRs and non-VIRs patterns were also observed in the TSL (See Additional file: 1 Figure S6). Thereafter, evolutionary information obtained from PSI-BLAST was used for the discrimination between VIRs and non-VIRs. Applying different machine learning algorithms of WEKA revealed that IBk method achieved maximum 50.70% sensitivity, 96.91% specificity, 92.71% accuracy and 0.52 MCC. SVM achieved highest 0.53 MCC with 52.19% sensitivity, 96.79% specificity and 92.73% accuracy. At the −0.8 thresholds level SVM achieved 78.52% sensitivity, 78.61% specificity, 78.60% accuracy and 0.37 MCC. Performances of all applied classifiers are provided in Table 1. As shown in Receiver Operating Curve (ROC) graph, binary (SVM), PSSM (IBk) and PSSM (SVM) achieved 0.74, 0.74 and 0.87 Area under curve (AUC) values, respectively (Figure 2). The performance increased significantly when PSSM was used as input instead of the binary patterns approach.

**Table 1:** **Prediction performance of different classifiers for vitamin**-**interacting residues** (**VIRs**)

**Feature**	** Classifier**	**SN**	**SP**	**ACC**	**MCC**
Binary	SVM (Threshold = −0.8)	68.57 ± 0.60	64.88 ± 0.18	65.22 ± 0.21	0.20 ± 0.00
	SVM (Threshold = −0.5)	29.53 ± 0.83	94.71 ± 0.16	88.78 ± 0.15	0.27 ± 0.01
	BayesNet	54.76 ± 1.44	69.64 ± 0.99	68.29 ± 0.85	0.15 ± 0.01
	ComplementNaiveBayes	67.57 ± 0.90	65.16 ± 0.29	65.38 ± 0.33	0.19 ± 0.01
	NaiveBayes	35.65 ± 0.85	89.52 ± 0.22	84.62 ± 0.18	0.22 ± 0.01
	NaiveBayesMultinomial	40.08 ± 1.04	87.67 ± 0.24	83.35 ± 0.24	0.22 ± 0.01
	IBk	26.67 ± 0.76	93.83 ± 0.11	87.73 ± 0.15	0.22 ± 0.01
	RandomForest	35.48 ± 0.78	79.13 ± 0.36	75.17 ± 0.31	0.10 ± 0.01
PSSM	**SVM** (**Threshold** = −**0**.**8**)	**78**.**52** ± **0**.**64**	**78**.**61** ± **0**.**34**	**78**.**60** ± **0**.**32**	**0**.**37** ± **0**.**01**
	*SVM* (*Threshold* = −*0*.*1*)	*52*.*19* ± *1*.*01*	*96*.*79* ± *0*.*03*	*92*.*73* ± *0*.*11*	*0*.*53* ± *0*.*01*
	BayesNet	67.41 ± 0.24	64.20 ± 0.06	64.49 ± 0.05	0.19 ± 0.00
	ComplementNaiveBayes	61.21 ± 0.58	78.06 ± 0.23	76.53 ± 0.19	0.26 ± 0.00
	NaiveBayes	67.64 ± 0.37	65.48 ± 0.11	65.68 ± 0.09	0.20 ± 0.00
	NaiveBayesMultinomial	54.91 ± 0.94	83.52 ± 0.21	80.92 ± 0.16	0.28 ± 0.01
	IBk	50.70 ± 0.90	96.91 ± 0.06	92.71 ± 0.08	0.52 ± 0.01
	RandomForest	61.54 ± 0.64	81.52 ± 0.12	79.70 ± 0.11	0.30 ± 0.01

*Bold value indicates highest performance with balanced sensitivity and specificity.

**Italic value indicates performance with highest MCC.

The values of standard errors are also given with performances.

#### Prediction of vitamin A interacting residues (VAIRs)

**Figure 3:**
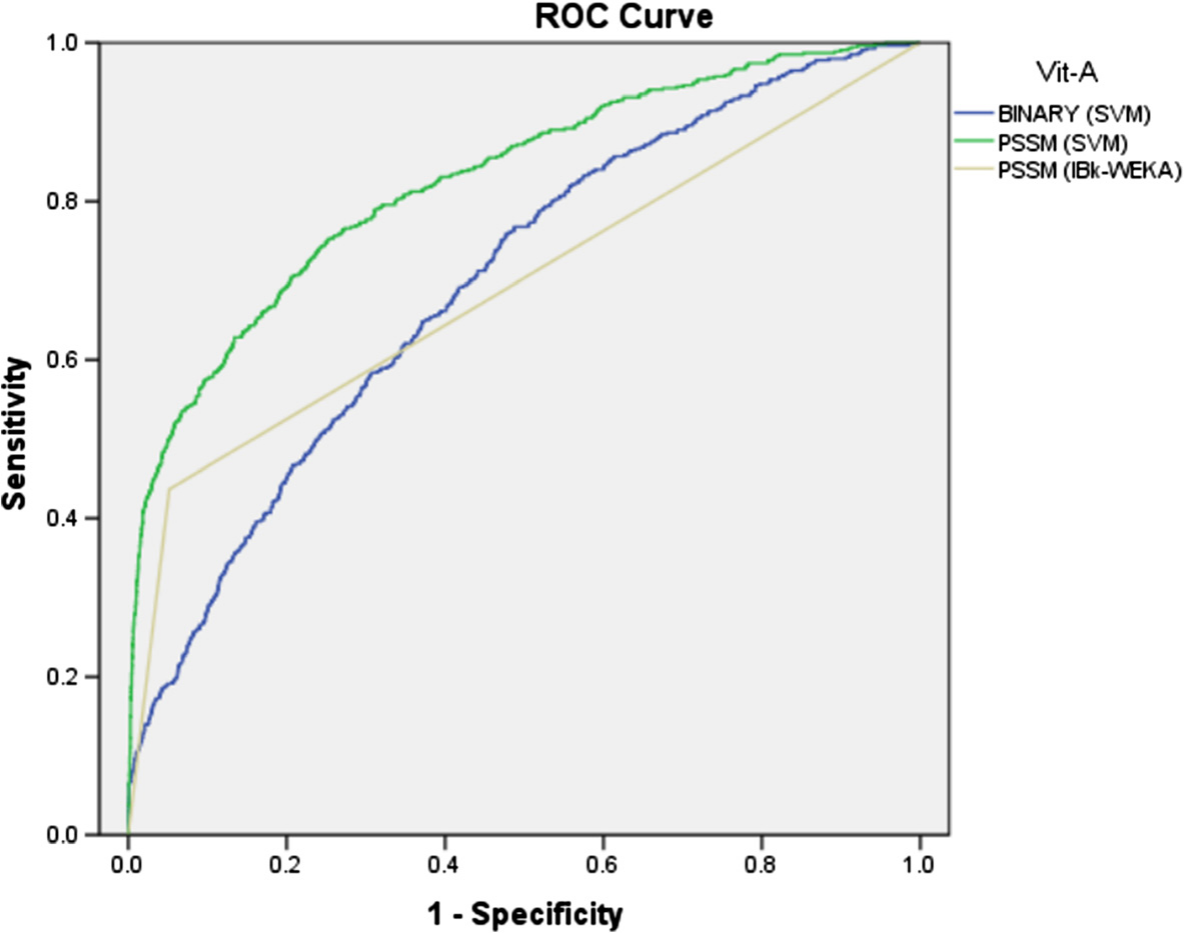
The ROC plot of the performance of different approaches for prediction of VAIRs.

We also developed prediction method for the VAIRs. The TSL of sliding patterns showed that Phe, Ile, Leu, Val and Trp were more abundant in VAIRs than in non-VAIRs (See Additional file 1: Figure S7). These patterns were converted into the binary profile of patterns in order to develop the SVM-based prediction model. This model achieved 61.92% sensitivity, 65.09% specificity, 64.80% accuracy and 0.16 MCC. The IBk based prediction model of PSSM achieved maximum 44.05% sensitivity, 94.65% specificity, 90.05% accuracy and 0.39 MCC. SVM based PSSM approach achieved highest MCC of 0.48 with 42.75% sensitivity, 97.51% specificity and 92.54% accuracy. At the −0.8 thresholds level SVM achieved balanced performance of 72.70% sensitivity, 76.89% specificity, 76.51% accuracy and 0.32 MCC. Table 2 shows performances of all applied classifiers. As shown in ROC graph, binary (SVM), PSSM (IBk) and PSSM (SVM) achieved 0.70, 0.70 and 0.83 AUC values, respectively (Figure 3). The PSSM based approach enhanced the prediction performance with SVM.

**Table 2:** **Prediction performance of different classifiers for vitamin A**-**interacting residues** (**VAIRs**)

**Feature**	** Classifier**	**SN**	**SP**	**ACC**	**MCC**
Binary	SVM (Threshold = −0.8)	61.92 ± 2.63	65.09 ± 0.43	64.80 ± 0.35	0.16 ± 0.02
	SVM (Threshold = −0.1)	7.43 ± 1.18	99.66 ± 0.10	91.28 ± 0.08	0.21 ± 0.02
	BayesNet	14.50 ± 2.11	94.30 ± 0.20	87.04 ± 0.22	0.10 ± 0.02
	ComplementNaiveBayes	62.09 ± 0.50	65.97 ± 0.22	65.61 ± 0.20	0.17 ± 0.00
	NaiveBayes	32.53 ± 0.99	86.43 ± 0.22	81.53 ± 0.27	0.15 ± 0.01
	NaiveBayesMultinomial	60.23 ± 0.82	67.94 ± 0.16	67.24 ± 0.15	0.17 ± 0.01
	IBk	31.41 ± 2.27	89.80 ± 0.20	84.49 ± 0.19	0.19 ± 0.02
	RandomForest	36.07 ± 2.03	78.38 ± 0.16	74.54 ± 0.30	0.10 ± 0.01
PSSM	**SVM** (**Threshold** = −**0**.**8**)	**72**.**70** ± **2**.**87**	**76**.**89** ± **0**.**25**	**76**.**51** ± **0**.**37**	**0**.**32** ± **0**.**02**
	*SVM* (*Threshold* =*0*.*0*)	*42*.*75* ± *1*.*08*	*97*.*51* ± *0*.*10*	*92*.*54* ± *0*.*13*	*0*.*48* ± *0*.*01*
	BayesNet	57.25 ± 1.21	69.54 ± 0.52	68.42 ± 0.48	0.16 ± 0.01
	ComplementNaiveBayes	59.30 ± 1.23	66.96 ± 0.33	66.26 ± 0.26	0.16 ± 0.01
	NaiveBayes	63.03 ± 1.65	69.09 ± 0.46	68.54 ± 0.56	0.19 ± 0.01
	NaiveBayesMultinomial	55.77 ± 1.32	70.95 ± 0.21	69.57 ± 0.26	0.17 ± 0.01
	IBk	44.05 ± 0.49	94.65 ± 0.34	90.05 ± 0.27	0.39 ± 0.01
	RandomForest	24.17 ± 0.80	99.31 ± 0.08	92.49 ± 0.06	0.41 ± 0.01

*Bold value indicates highest performance with balanced sensitivity and specificity.

**Italic value indicates performance with highest MCC.

The values of standard errors are also given with performances.

#### Prediction of vitamin B interacting residues (VBIRs)

**Figure 4:**
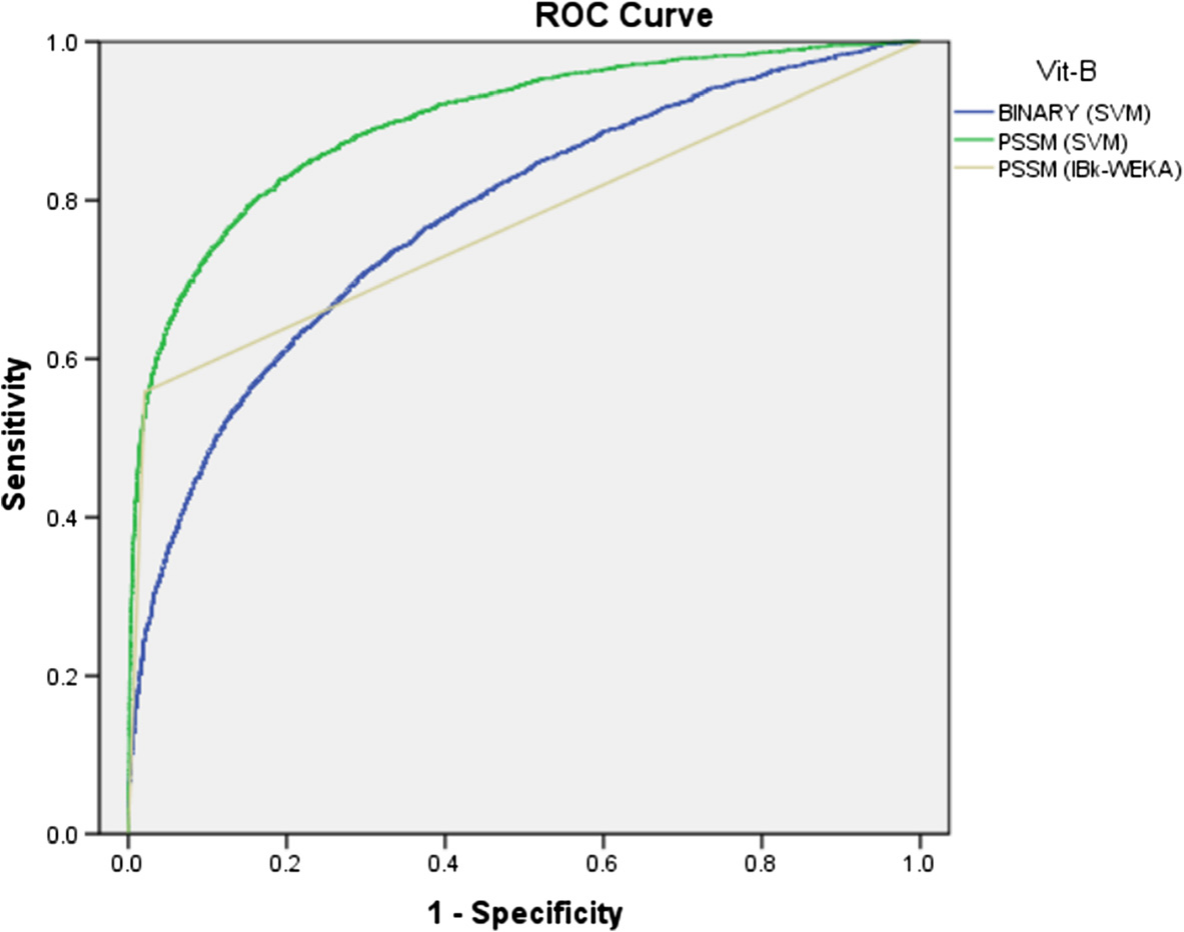
The ROC plot of the performance of different approaches for prediction of VBIRs.

The TSL analysis of VBIRs and non-VBIRs showed that Gly, His, Asn, Ser, Thr, Trp and Tyr were more abundant in VBIRs (See Additional file: 1 Figure S8). The SVM-based prediction model was developed using binary patterns and achieved 73.22% sensitivity, 67.00% specificity, 67.57% accuracy and 0.24 MCC. The IBk based prediction model of PSSM achieved maximum 56.74% sensitivity, 98.04% specificity, 94.28% accuracy and 0.62 MCC. SVM based PSSM approach achieved highest 0.61 MCC with 55.57% sensitivity, 98.04% specificity and 94.18% accuracy. At the −0.8 thresholds level SVM achieved 81.39% sensitivity, 81.77% specificity, 81.73% accuracy and 0.43 MCC. Performances of all applied classifiers are provided in Table 3. As shown in ROC graph, binary (SVM), PSSM (IBk) and PSSM (SVM) achieved 0.78, 0.77 and 0.90 AUC values, respectively (Figure 4). The overall performance increased by PSSM profiles based model, in compare to binary patterns based approaches.

**Table 3:** **Prediction performance of different classifiers for vitamin B**-**interacting residues** (**VBIRs**)

**Feature**	** Classifier**	**SN**	**SP**	**ACC**	**MCC**
Binary	SVM (Threshold = −0.8)	73.22 ± 0.36	67.00 ± 0.49	67.57 ± 0.47	0.24 ± 0.00
	SVM (Threshold = −0.6)	30.36 ± 0.62	96.69 ± 0.12	90.66 ± 0.11	0.33 ± 0.01
	BayesNet	63.25 ± 0.56	66.23 ± 0.73	65.96 ± 0.62	0.18 ± 0.00
	ComplementNaiveBayes	68.69 ± 0.52	68.51 ± 0.23	68.52 ± 0.18	0.23 ± 0.00
	NaiveBayes	37.74 ± 0.90	90.45 ± 0.23	85.66 ± 0.14	0.25 ± 0.01
	NaiveBayesMultinomial	44.22 ± 0.43	87.54 ± 0.24	83.60 ± 0.19	0.25 ± 0.00
	IBk	30.81 ± 0.71	93.33 ± 0.17	87.65 ± 0.14	0.24 ± 0.01
	RandomForest	39.33 ± 1.08	79.36 ± 0.37	75.72 ± 0.36	0.13 ± 0.01
PSSM	**SVM** (**Threshold** = −**0**.**8**)	**83**.**33** ± **0**.**36**	**80**.**51** ± **0**.**13**	**80**.**77** ± **0**.**14**	**0**.**42** ± **0**.**00**
	*SVM* (*Threshold* =*0*.*1*)	*55*.*57* ± *0*.*63*	*98*.*04* ± *0*.*10*	*94*.*18* ± *0*.*09*	*0*.*61* ± *0*.*01*
	BayesNet	71.65 ± 1.13	66.14 ± 0.08	66.64 ± 0.10	0.23 ± 0.01
	ComplementNaiveBayes	63.90 ± 1.26	81.73 ± 0.28	80.11 ± 0.22	0.32 ± 0.01
	NaiveBayes	72.28 ± 1.22	66.44 ± 0.09	66.97 ± 0.12	0.23 ± 0.01
	NaiveBayesMultinomial	21.22 ± 0.69	98.88 ± 0.03	91.82 ± 0.06	0.34 ± 0.01
	*IBk*	*56*.*74* ± *0*.*80*	*98*.*04* ± *0*.*07*	*94*.*28* ± *0*.*11*	*0*.*62* ± *0*.*01*
	RandomForest	39.16 ± 0.56	97.74 ± 0.09	92.41 ± 0.10	0.46 ± 0.01

*Bold value indicates highest SVM performance with balanced sensitivity and specificity.

**Italic value indicates SVM/IBk performance with highest MCC.

The values of standard errors are also given with performances.

#### Prediction of pyridoxal-5-phosphate interacting residues (PLPIRs)

**Figure 5:**
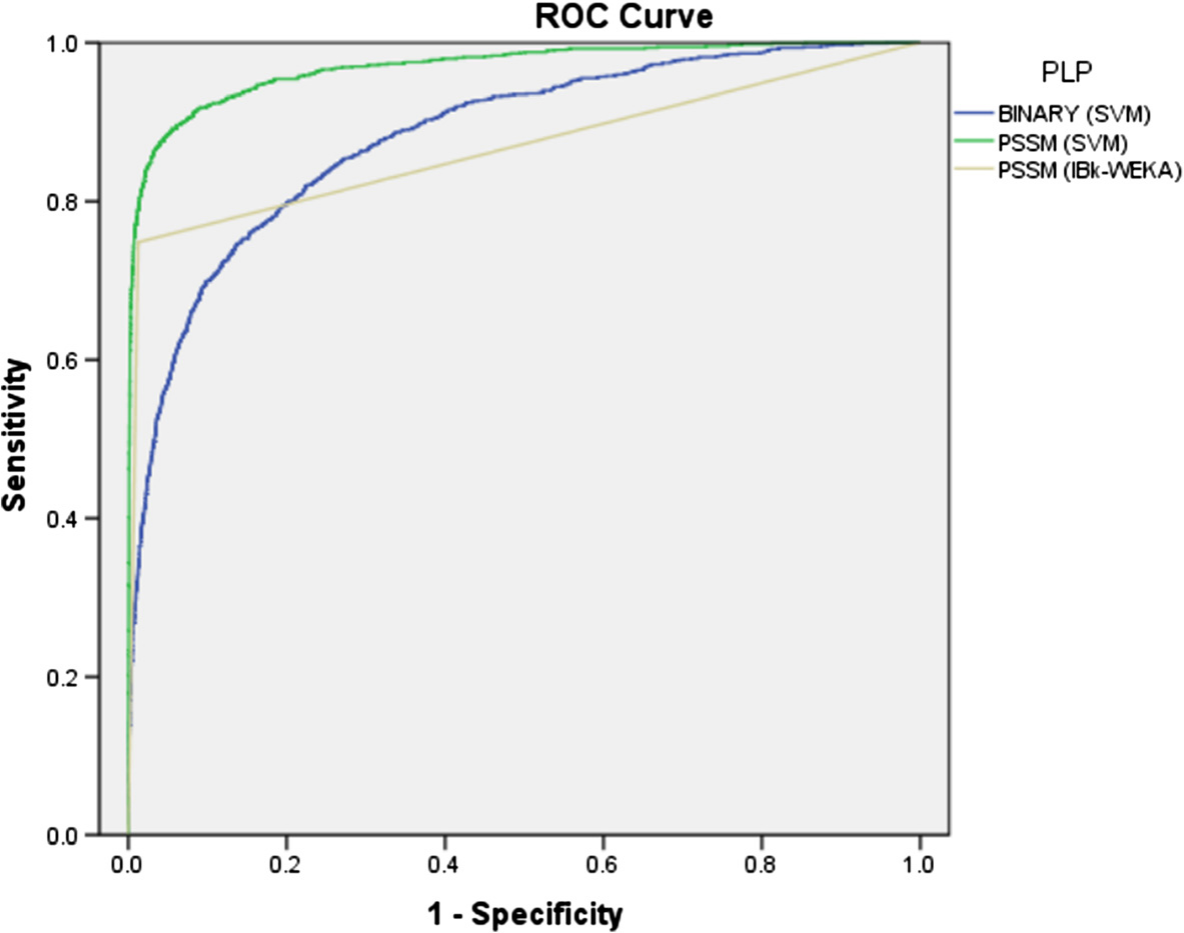
The ROC plot of the performance of different approaches for prediction of PLPIRs.

The compositional and TSL analysis of PLPIRs and non-PLPIRs found that Gly, His, Asn, Ser, Thr and Tyr were more abundant in PLPIRs (See Additional file 1: Figure S9). The binary patterns (17-length windows) based prediction model achieved 77.02% sensitivity, 83.17% specificity, 82.62% accuracy and 0.42 MCC. The IBk based PSSM approach achieved 76.10% sensitivity, 98.80% specificity, 96.74% accuracy and 0.79 MCC whereas SVM based achieved highest 0.81 MCC with 79.76% sensitivity, 98.62% specificity, 96.91% accuracy. At the −0.7 thresholds level SVM achieved 79.76% sensitivity, 98.62% specificity, 96.91% accuracy and 0.81 MCC. As shown in ROC graph, binary (SVM), PSSM (IBk) and PSSM (SVM) achieved 0.88, 0.87 and 0.97 AUC values, respectively (Figure 5). Table 4 shows performances of all applied classifiers. Here also PSSM profile based evolutionary information enhanced the prediction performance of SVM model.

**Table 4:** **Prediction performance of different classifiers for PLP**-**interacting residues** (**PLPIRs**)

**Feature**	** Classifier**	**SN**	**SP**	**ACC**	**MCC**
Binary	SVM (Threshold = −0.7)	77.02 ± 0.72	83.17 ± 0.27	82.62 ± 0.28	0.42 ± 0.01
	SVM (Threshold = −0.5)	54.76 ± 1.34	95.81 ± 0.14	92.08 ± 0.18	0.51 ± 0.01
	BayesNet	41.76 ± 0.81	88.94 ± 0.49	84.65 ± 0.40	0.26 ± 0.01
	ComplementNaiveBayes	75.82 ± 1.74	77.14 ± 0.35	77.01 ± 0.23	0.34 ± 0.01
	NaiveBayes	52.20 ± 1.50	91.18 ± 0.17	87.64 ± 0.20	0.37 ± 0.01
	NaiveBayesMultinomial	59.25 ± 1.06	88.51 ± 0.19	85.85 ± 0.19	0.38 ± 0.01
	IBk	40.02 ± 1.24	96.31 ± 0.20	91.19 ± 0.21	0.41 ± 0.01
	RandomForest	52.93 ± 1.09	80.03 ± 0.71	77.56 ± 0.65	0.23 ± 0.01
PSSM	**SVM** (**Threshold** = −**0**.**7**)	**90**.**20** ± **1**.**04**	**92**.**61** ± **0**.**18**	**92**.**40** ± **0**.**13**	**0**.**67** ± **0**.**00**
	*SVM* (*Threshold* = −*0*.*1*)	*79*.*76* ± *0*.*92*	*98*.*62* ± *0*.*13*	*96*.*91* ± *0*.*11*	*0*.*81* ± *0*.*01*
	BayesNet	77.66 ± 0.83	77.71 ± 0.35	77.70 ± 0.30	0.36 ± 0.01
	ComplementNaiveBayes	76.28 ± 1.46	89.09 ± 0.54	87.93 ± 0.45	0.50 ± 0.01
	NaiveBayes	79.40 ± 0.76	80.36 ± 0.35	80.28 ± 0.27	0.40 ± 0.00
	NaiveBayesMultinomial	43.96 ± 0.67	98.16 ± 0.08	93.25 ± 0.07	0.52 ± 0.01
	IBk	76.10 ± 0.82	98.80 ± 0.06	96.74 ± 0.08	0.79 ± 0.01
	RandomForest	62.27 ± 1.76	98.02 ± 0.12	94.78 ± 0.20	0.66 ± 0.01

*Bold value indicates highest performance with balanced sensitivity and specificity.

**Italic value indicates performance with highest MCC.

The values of standard errors are also given with performances.

### Performance of balanced datasets

We also developed the SVM-based prediction models on the balanced datasets using both binary and PSSM approaches. The binary approach achieved 0.32, 0.24, 0.37 and 0.52 MCC for VIRs, VAIRs, VBIRs and PLPIRs respectively (Table 5). The PSSM approach improved the prediction performance significantly and achieved 0.53, 0.47, 0.63 and 0.80 MCC for VIRs, VAIRs, VBIRs and PLPIRs respectively (Table 5).

**Table 5:** **SVM**-**based prediction performances for four different types of prediction methods using equal positive and negative instances**

**Prediction**	**Binary approach**				**PSSM approach**			
	**Sensitivity**	**Specificity**	**Accuracy**	**MCC**	**Sensitivity**	**Specificity**	**Accuracy**	**MCC**
VIRs	65.98 ± 0.85	65.85 ± 0.52	65.91 ± 0.60	0.32 ± 0.01	75.80 ± 0.35	77.07 ± 0.69	76.43 ± 0.47	0.53 ± 0.01
VAIRs	62.09 ± 2.01	61.87 ± 2.92	61.99 ± 1.30	0.24 ± 0.03	73.25 ± 2.43	73.83 ± 0.95	73.54 ± 1.47	0.47 ± 0.03
VBIRs	68.55 ± 0.75	68.37 ± 0.83	68.47 ± 0.44	0.37 ± 0.01	80.08 ± 0.61	82.49 ± 0.79	81.29 ± 0.23	0.63 ± 0.01
PLPIRs	76.74 ± 1.73	74.91 ± 1.42	75.82 ± 1.32	0.52 ± 0.03	89.85 ± 0.87	89.85 ± 1.16	89.84 ± 0.70	0.80 ± 0.01

The values of standard errors are also given with performances.

### Performance on the independent datasets

Four different independent datasets, V-IND-46, VA-IND-15, VB-IND-27 and PLP-IND-16, containing 46, 15, 27 and 16 protein sequences and utilized for the evaluation of VIRs, VAIRs, VBIRs and PLPIRs prediction methods, were used. We used SVM-based binary approach, calculated performances at already optimized threshold level (by 5-fold cross validation of main-dataset) and achieved highest 0.19, 0.23, 0.20 and 0.30 MCC for the prediction of VIRs, VAIRs, VBIRs and PLPIRs respectively (See Additional file 1: Table S1). The performance enhanced significantly while using PSSM approach and achieved highest 0.38, 0.37, 0.35 and 0.63 MCC for the prediction of VIRs, VAIRs, VBIRs and PLPIRs respectively (Table 6).

**Table 6:** **SVM**-**based prediction performances** (**at the default threshold**) **of PSSM approach on the different independent datasets**

**S**.**No**.	**Prediction**	**Dataset**	**Threshold**	**Sensitivity**	**Specificity**	**Accuracy**	**MCC**
1	VIRs	V-IND-46	−**0**.**8**	**73**.**70**	**71**.**98**	**72**.**07**	**0**.**22**
			−*0*.*1*	*41*.*74*	*96*.*63*	*93*.*72*	*0*.*38*
2	VAIRs	VA-IND-15	−**0**.**8**	**73**.**48**	**72**.**87**	**72**.**93**	**0**.**31**
			*0*.*0*	*30*.*39*	*97*.*22*	*89*.*77*	*0*.*37*
3	VBIRs	VB-IND-27	−**0**.**8**	**83**.**05**	**68**.**76**	**69**.**40**	**0**.**23**
			*0*.*1*	*49*.*40*	*94*.*49*	*92*.*47*	*0*.*35*
4	PLPIRs	PLP-IND-16	−**0**.**7**	**84**.**15**	**83**.**22**	**83**.**26**	**0**.**33**
			−*0*.*1*	*65*.*85*	*98*.*40*	*97*.*10*	*0*.*63*

*Bold value indicates performance at the optimized threshold level of balanced sensitivity and specificity.

**Italic value indicates performance at the optimized threshold level of highest MCC.

### Surface accessibility based prediction

Most of binding residues reside inside the surface pockets and predicting these pockets is therefore important. For these predictions, it is required to firstly predict the surface accessibility (SA) of each residue from the protein sequence. Therefore, we used SARpred method [51] for the prediction of surface accessibility of all residues. On the basis of these surface accessibility values, we tried to develop SVM-based models but as shown in the Additional file 1: Table S2 the performances were very poor on the realistic dataset. On the balanced dataset, SA-based approach achieved 0.15, 0.08, 0.22 and 0.30 MCC for the prediction of VIRs, VAIRs, VBIRs and PLPIRs respectively. The major limitation of this approach was that surface accessibility feature itself was predicted from the protein sequences. The results were showing that only PLP-interacting residues could be predicted (MCC 0.30) with surface accessibility while other predictors performed poorly (See Additional file 1: Table S2). The performance of PLPIRs predictor was better than the performance from this study. This may be because of the presence of more than one ligand in the other predictors (VIR, VAIR, VBIR). There may be chances that binding pockets were very different for each ligand and therefore difficult to model. Sometime, it is better to combine more than two features, in order to achieve good prediction results. In-spite of a combined PSSM-surface accessibility approach, we were unable to achieve any improvement in performance measures over the single PSSM-based approach for both the realistic and balanced datasets (See Additional file 1: Table S2). These results suggest that PSSM-based individual approach performances were as good as combined approach with both PSSM and surface accessibility features.

### Quality of PSSM profiles

The number of homology sequences can affect the quality of PSSM profiles; therefore it is important to check the quality of PSSM profiles. Earlier this type of analysis has been done for the prediction of DNA-binding proteins in the DNAbinder method [27]. The number of homology sequences depends on total number of the protein sequences in the database. We used PSI-BLAST program for the default parameters with 3 iterations and checked the prediction performance on the different independent datasets. The independent datasets of VIRs, VAIRs, VBIRs and PLPIRs are V-IND-46, VA-IND-15, VB-IND-27 and PLP-IND-16 and containing 46, 15, 27, and 16 protein sequences respectively. The prediction performances (at default threshold level) of different independent datasets are shown in the Additional file 1: Table S3. As the total numbers of homology sequences were different for each query sequence; by default it varied from the 0–500 sequences. On the basis of total PSI-BLAST hits, we divided each dataset into five different categories (overall 0–500, 0–10, 11–100, 101–400 and 401–500). As mentioned in the Additional file 1: Table S3, it was observed that performances increased with the increment of number of homolog sequences. Prediction performances were poor for the 0–10 and 11–100 ranges of query sequences in all four cases whereas average for the 101–400 range and good for the 401–500 homolog sequences.

These results suggested that the quality of PSSM profiles depends on the number of homolog sequences. In most of cases, the major fraction of sequences ranged between 401–500 (PSI-BLAST hit range). The overall performances of simple binary-based approach (Additional file 1: Table S1) were higher than the PSSM-based prediction that had range values between 0–10 (Additional file 1: Table S3).

## Methods

### Datasets

In this study, we collected data from SuperSite documentation [52] and extracted 1061 PDB IDs of protein having contact with vitamins in PDB. We downloaded the sequence of all chains of these PDB Ids from Protein Data Bank [53]. In next step, we used these PDB IDs in Ligand Protein Contact (LPC) web-server [54] and get total 2720 chains that interact with vitamins with their corresponding interacting residues and its position. We used a cut-off of 5.0 Å to define the vitamin interacting residues. A residue was considered to be vitamin-interacting if the closest distance between atoms of the protein and the partner vitamin was within the cut-off (5 Å). The 25% non-redundant dataset of protein chains was created by using BLASTCLUST and finally retrieved a total 187 interacting chains with a total 3004 vitamin-interacting residues (VIRs) and remaining all residues are non-vitamin-interacting residues (non-VIRs). This step was repeated for the dataset development of vitamin A, vitamin B and PLP (vitamin B6-derived) interacting residue prediction and retrieved 538, 2207 and 1092 interacting residues in 31, 141 and 71 chains respectively. The interacting and non-interacting residues were used as positive and negative instances respectively. The number of non-interacting residues was very large than interacting residues so we have randomly picked up 10 times more non-interacting than interacting residues in order to create realistic dataset. The balanced datasets of equal positive and negative were also created, where equal numbers of random negative instances was taken from the total negative window patterns.

We created four different independent datasets: V-IND-46, VA-IND-15, VB-IND-27 and PLP-IND-16 of the 46, 15, 27 and 16 protein sequences for the prediction of VIRs, VAIRs, VBIRs and PLPIRs respectively. All these datasets were 25% non-redundant and all sequences of these independent datasets were less than 25% similar than sequences of main datasets.

### Window patterns and size

We generated sliding (overlapping) patterns of 17-residue size, for each interacting chain sequence. In past, several studies have adopted this strategy for the interacting residue tools development [40,45]. If the central residue of pattern was interacting, then we classified the pattern as interacting or positive pattern; otherwise it was termed as non-interacting or negative pattern. To generate the pattern corresponding to the terminal residues in a protein sequence, we have added (L-1)/2 dummy residue "X" at both terminals of protein (where L is the length of pattern). Here the length of pattern is 17 so we have added 8 "X" before N-terminal and 8 "X" after C-terminal, in order to create equal number of patterns from sequence length.

### Binary profile of patterns

These positive and negative patterns were converted into the binary patterns and all amino acids represented by a vector of 21 dimensions (e.g. Ala by 1,0,0,0,0,0,0,0,0,0,0,0,0,0,0,0,0,0,0,0,0; Cys by 0,1,0,0,0,0,0,0,0,0,0,0,0,0,0,0,0,0,0,0,0), which contained 20 standard amino acids and one dummy amino acid “X”. We used these profiles as an input data of various machine-learning algorithms.

### Position-Specific Scoring Matrix (PSSM)

We performed PSI-BLAST (position-specific iterative BLAST) search (default parameter) against the non-redundant (NR) database available at Swiss-Prot [55]. After three iterations, PSI-BLAST generated the PSSM profiles with the highest score from multiple alignments of the high-scoring hits by calculating the position-specific scores for each position in the alignments. The PSSM profile contains the occurrence probability of all amino acids at each position along with insertion/deletion and provides the evolutionary information for all amino acids. The final PSSM was normalized using a sigmoid function.

### Surface accessibility

We calculated surface accessibility value for each residue of the all sequences using SARpred method [51]. We normalized these values (between minimum to maximum) and assigned a value for the each residue of the 17-length window patterns. We used these 17 input features for the SVM-based prediction of VIRs, VAIRs, VBIRs and PLPIRs. In the hybrid approach with PSSM, we combined these 17 input features with the PSSM features.

### Support vector machine

In this study, a highly successful machine learning technique termed as a *Support Vector Machine* (SVM) was used. SVM is a machine-learning tool and based on the structural risk minimization principle of statistics learning theory. SVMs are a set of related supervised learning methods used for classification and regression [56]. The user can choose and optimize number of parameters and kernels (e.g. Linear, polynomial, radial basis function and sigmoidal) or any user-defined kernel. In this study, we implemented SVM^light^ Version 6.02 package [57] of SVM and machine learning was carried out using three different (linear, polynomial and radial basis function) kernels. SVM takes a set of fixed length input features, along with their output, which is used for training of model. After training, learned model can be used for prediction of unknown examples [58]. We optimized different parameters and kernels for all approaches and developed efficient prediction tools.

### WEKA package

WEKA is a large collection of various machine-learning algorithms as single package [59]. We applied WEKA 3.6.4 version, which integrates different classifiers such as BayesNet, NaiveBayes, ComplementNaiveBayes, NaiveBayesMultinomial, RandomForest and IBk. All algorithms have been applied and optimized for different prediction tool development.

### Five-fold cross validation

The validation of any prediction method is very essential part. In this study, we have used a five-fold cross-validation technique [60] for training, testing and evaluating our prediction methods. The protein sequences/patterns of positive and negative instances were randomly divided into five parts. Each of these five sets consists of one-fifth of positive and one-fifth of negative instances. In this technique, the training and testing was carried out five times, each time using one distinct set for testing and the remaining four sets for training.

### Evaluation parameters

To assess the performance of various modules developed in this study, we calculated the sensitivity, specificity, accuracy and Matthew's correlation coefficient (MCC). These calculations were routinely used in these types of prediction-based studies [61,62]. These parameters were calculated using following equations (14):

(1) $$\text{Sensitivity}=\frac{\text{TP}}{\text{TP}+\text{FN}}\times 100$$

(2) $$\text{Specificity}=\frac{\text{TN}}{\text{TN}+\text{FP}}\times 100$$

(3) $$\text{Accuracy}=\frac{\text{TP}+\text{TN}}{\text{TP}+\text{FP}+\text{TN}+\text{FN}}\times 100$$

(4) $$\text{MCC}=\frac{\left(\text{TP}\times \text{TN}\right)-\left(\text{FP}\times \text{FN}\right)}{\sqrt{\left(\text{TP}+\text{FP}\right)\left(\text{TP}+\text{FN}\right)\left(\text{TN}+\text{FP}\right)\left(\text{TN}+\text{FN}\right)}}$$

Where TP and TN are correctly predicted positive and negative examples, respectively. Similarly, FP and FN are wrongly predicted positive and negative examples respectively.

The standalone version of *VitaPred* gives prediction results with probability score instead of SVM score. We have calculated probability score by using following equation –

(5) $$\text{Probability}\;\text{score}=\frac{\text{SVM}\;\text{score}+1.5}{3}\times 9$$

We rescaled the SVM scores with maximum 1.5 and minimum −1.5, where more than 1.5 and less than −1.5 both scores were used as 1.5 and −1.5 respectively. The probability score varies from 0–9 for each residue of protein sequence. The probability scores ranges between 0–4 and 5–9 predicted as non-interacting and interacting residues respectively at default 0.0 thresholds.

The five fold cross-validation technique created five test sets and calculated performance for each test set. The final performance of prediction model is an average performance of these five test sets. In this average performance, we also calculated standard error of the performance of these five test set. MCC is considered to the most robust parameters for the evaluation of any prediction method [63]. The MCC value ranges between +1 to −1. The MCC value of 1 corresponds to a perfect prediction, whereas 0 corresponds to a completely random prediction. The −1 MCC value indicates total disagreement between prediction and actual examples. The evaluation parameters of SVM performances are threshold-dependent and require parameters/kernels optimization for the better results. The complete optimization of all parameters is key step in SVM based machine learning. We manually optimized all parameters and selected the highly performed prediction models for different tasks. In order to have a threshold independent evaluation of our method, we also created ROC and calculated AUC value for the threshold independent evaluation using SPSS statistical package.

### Two sample logo (TSL)

In this study, we have created Two Sample Logo (http://www.twosamplelogo.org/) for the graphical representation of positive and negative patterns [64]. It is a web-based application to calculate and visualize position-specific differences between positive and negative samples.

### Web-server

A user-friendly web-server *VitaPred* developed for the prediction of VIRs, VAIRs, VBIRs and PLPIRs in protein sequence. The VitaPred is freely available from http://crdd.osdd.net/raghava/vitapred/ web-address. It requires protein sequence in standard FASTA format. There are four different type of options provided for the prediction of VIRs, VAIRs, VBIRs and PLPIRs. We have also provided our datasets and other supplementary materials, which were used for the development of *VitaPred* web-server.

### Standalone version of VitaPred

In the era of genomics, it is essential to develop computational tools for the huge amount of sequence data. We have developed standalone version of *VitaPred* by using Visual Basic .NET technologies. This is available from the site of web-server. User can download and install it in their system. This software gives the results with probability scores (Equation 5) for each residue of protein sequences. The multiple sequences can efficiently proceed with this software.

## Discussion

The experimental determination of vitamin binding sites is very difficult task because of their complex chemical nature, and the fact that they are often made in very small amounts, making detection of the enzyme activities and intermediates difficult [4]. So there is a need to develop alternate technique, such as computational techniques for predicting vitamin-binding sites in a protein. The comparative analysis of different ligands with VIR (Additional file 1: Figure S6) such as ATP (Additional file 1: Figure S1), GTP (Additional file 1: Figure S2), NAD (Additional file 1: Figure S3), FAD (Additional file 1: Figure S4) and mannose (Additional file 1: Figure S5) revealed that each ligand has different protein-binding patterns (See all Figures in Additional file 1). Thus, it is important to develop a separate vitamin-interacting residues prediction tool.

We have used available structural information (knowledge-based) for the prediction model development using different machine learning algorithms. The structural information of protein-vitamin complexes extracted from SuperSite [52]. We found total 1061 protein-vitamin complexes, in which 181 and 843 complexes proteins are bind with vitamin A and B respectively. Out of these total 843 complexes of vitamin B binding complexes, 553 are bind to vitamin B(6)-derived pyridoxal 5'-phosphate (PLP) binding protein. The structural availability of vitamin C, D, E and K binding protein complexes are very low in PDB. Thus, we have developed four different methods for the prediction of VIRs, VAIRs, VBIRs and PLPIRs. We identified interacting and non-interacting residues using Ligand Protein Contact (LPC) web server [54]. The interacting residues analysis suggested that Phe, Gly, His, Ser, Thr, Trp and Tyr amino acids are preferred in the vitamin binding pockets of Vitamin Binding Proteins (VBPs) (Figures 1). The preference of interacting and neighboring residues is vitamin class-specific (See Additional file 1: Figure S6-S9). In the past, it has been shown in some studies that multiple sequence alignment based evolutionary information provides more comprehensive detail about the protein instead of single sequence [51,65]. Thus, all sequences of datasets were created into PSSM profiles and used for the prediction tool development. The comparative analysis between vitamin A and B interacting sites showed that Phe, Ile, Leu, Val and Trp are abundant in VAIRs whereas Asp, Glu, Gly, His, Lys, Asn. Arg, Ser and Thr are abundant in VBIRs (Figure 1, See Additional file 1: Figure S7-S8). The vitamin B(6)-derived pyridoxal 5'-phosphate (PLP) is the cofactor of enzymes catalyzing a large variety of chemical reactions (more than 140 enzymes are PLP-dependent) mainly involved in amino acid metabolism [66]. According to the Enzyme Commission, about 4% of enzyme-catalyzed reactions are PLP-dependent (EC; http://www.chem.qmul.ac.uk/iubmb/enzyme/). Therefore, it was very important to develop a separate prediction model for the PLPIRs in protein sequence. The PSSM based approach achieved maximum performance for PLPIRs because of separate model for a single PLP molecule. The VIRs, VAIRs and VBIRs modules performed relatively low because each class comprises more than one molecule. It means the overall prediction performance of VIRs is an approximately combined performance of all vitamins.

The performances of all the used classifiers are also provided in the Tables 1, 2, 3, and 4. It was observed that PSSM feature based SVM classifier performed better in all cases, in term of balancing between sensitivity and specificity. The threshold-independent performance of SVM is better than IBk for all modules (Figures 2, 3, 4 and 5). In the 5-fold cross validation, we got total five prediction performances corresponding to five test sets and computed average performance and standard error (SE) from these 5 performances. In most of cases, we found low value of SE, which is variation in the performance over five sets (it is not performance of variation on individual protein/chain). As patterns were divided randomly in five sets so it is expected that performance in each set will be nearly same. In other words, low SE values show that distribution of patterns in sets is not biased. Moreover, SE is not affected by similarity between patterns or protein chains, as this SE only measures biasness in distribution of patterns in five sets.

The prediction performances on the different independent datasets show that these modules can predict interacting residues of all vitamin classes with reasonably good accuracy (Table 6). The quality of PSSM profiles were also investigated and found that protein sequences in our dataset have fairly high number of hits. Furthermore we also found PSSM approach based prediction performances increase with the increasing number of PSI-BLAST hits of the query sequence. As discussed, vitamins are crucial for the activation of many enzymes and crystal structures of many VBPs are unsolved. Furthermore, many vitamin-dependent enzymes have been used as a potential drug targets, thus residue level study of vitamin-interacting and non-interacting sites will be use for the further drug discovery processes.

## Conclusions

In order to assist the biologists in assigning the vitamin-interacting residues of VBPs, a systematic attempt has been made for predicting the vitamin-binding sites (VIRs, VAIRs, VBIRs and PLPIRs) from the amino acid sequence of VBPs. This study demonstrates that PSSM evolutionary information can be use to predict vitamin-binding sites in a protein sequence.

## Abbreviations

VBP: Vitamin Binding Protein; TSL: Two Sample Logo; VIR: Vitamin Interacting Residue; VAIR: Vitamin-A Interacting Residue; VBIR: Vitamin-B Interacting Residue; PLPIR: Pyridoxal-5-phosphate Interacting Residue; PLP: Pyridoxal-5-phosphate; SVM: Support Vector Machine; PSSM: Position-Specific Scoring Matrix; MCC: Matthew's correlation coefficient; ROC: Receiver Operating Curve; AUC: Area under curve.

## Competing interests

The authors declare that they have no competing interests.

## Authors’ contributions

BP and SG created dataset, developed the SVM models, created the backend web server and the front end user interface. BP developed windows-based standalone software *VitaPred* by using Visual Basic .NET technologies. GPSR conceived the project, coordinated it and refined the final manuscript drafted by BP and SG. All the authors have read and approved final manuscript.

## Supplementary Material

Additional file 1: Figure S1–S5 The TSL representation of sliding patterns (17-residues length) of ATP, GTP, NAD, FAD and mannose. The central residue (9^th^ position) is showing interacting (positive) and non-interacting (negative) residues. **Figure S6–S9.** The TSL representation of sliding patterns (17-residues length) for prediction of VIRs, VAIRs, VBIRs and PLPIRs. The central residue (9^th^ position) is showing VIRs (positive) and non-VIRs (negative). **Table S1.** SVM-based prediction performances of surface accessibility (SA) and Hybrid (PSSM + SA) approaches for four different types of prediction methods on both realistic and balanced datasets. The values of standard errors are also given with performances. **Table S2.** SVM-based prediction performances (at the default threshold) of PSSM approach; according to their total number PSI-BLAST hits of different independent datasets. **Table S3.** SVM-based prediction performances (at the default threshold) of binary approach on the different independent datasets. Click here for file
