# Supplementary material for: Prediction of vitamin interacting residues in a vitamin binding protein using evolutionary information
Source: BMC Bioinformatics. 2013 Feb 7;14:44. doi: 10.1186/1471-2105-14-44 (PMC3577447; doi:10.1186/1471-2105-14-44)
Supplement: Additional file 1: Figure S1–S5 — The TSL representation of sliding patterns (17-residues length) of ATP, GTP, NAD, FAD and mannose. The central residue (9th position) is showing interacting (positive) and non-interacting (negative) residues. Figure S6–S9. The TSL representation of sliding patterns (17-residues length) for prediction of VIRs, VAIRs, VBIRs and PLPIRs. The central residue (9th position) is showing VIRs (positive) and non-VIRs (negative). Table S1. SVM-based prediction performances of surface accessibility (SA) and Hybrid (PSSM + SA) approaches for four different types of prediction methods on both realistic and balanced datasets. The values of standard errors are also given with performances. Table S2. SVM-based prediction performances (at the default threshold) of PSSM approach; according to their total number PSI-BLAST hits of different independent datasets. Table S3. SVM-based prediction performances (at the default threshold) of binary approach on the different independent datasets. [file 1471-2105-14-44-S1.docx]

**Additional file 1**

**Title: Prediction of vitamin interacting residues in a vitamin binding protein using evolutionary information**


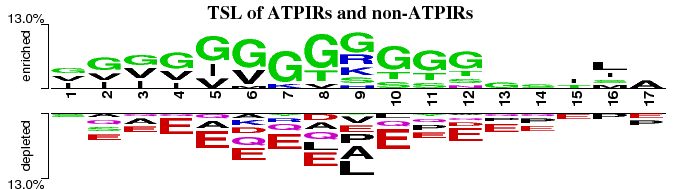


**Figure S1:** The TSL representation of sliding patterns (17-residues length) of ATP. The central residue (9^th^ position) is showing interacting (positive) and non-interacting (negative) residues.


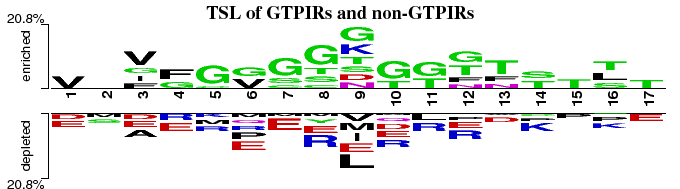


**Figure S2:** The TSL representation of sliding patterns (17-residues length) of GTP. The central residue (9^th^ position) is showing interacting (positive) and non-interacting (negative) residues.


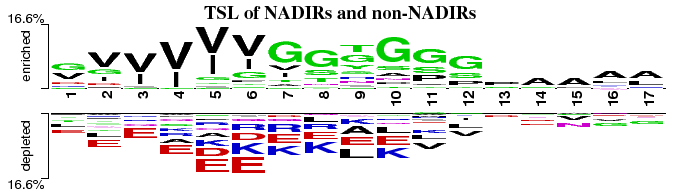


**Figure S3:** The TSL representation of sliding patterns (17-residues length) of NAD. The central residue (9^th^ position) is showing interacting (positive) and non-interacting (negative) residues.


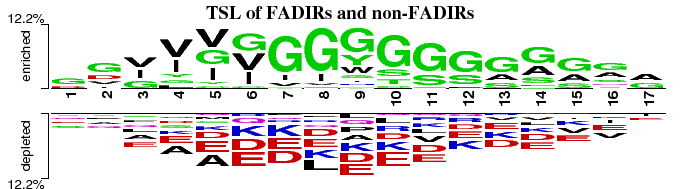


**Figure S4:** The TSL representation of sliding patterns (17-residues length) of FAD. The central residue (9^th^ position) is showing interacting (positive) and non-interacting (negative) residues.


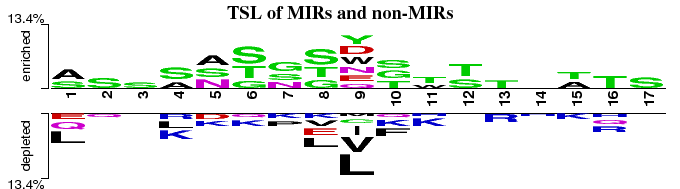


**Figure S5:** The TSL representation of sliding patterns (17-residues length) of mannose. The central residue (9^th^ position) is showing interacting (positive) and non-interacting (negative) residues.


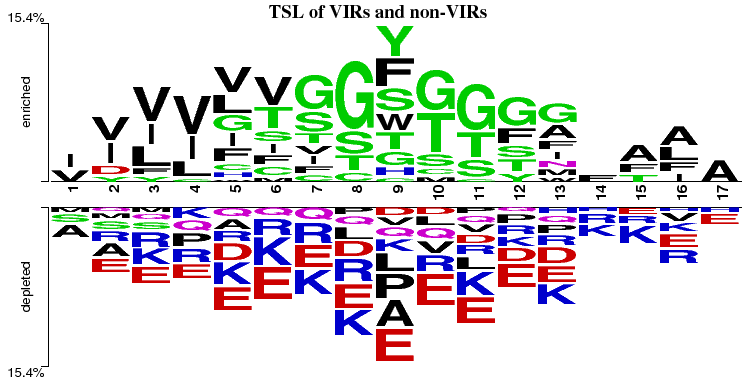


**Figure S6:** The TSL representation of sliding patterns (17-residues length) for prediction of VIRs. The central residue (9^th^ position) is showing VIRs (positive) and non-VIRs (negative).


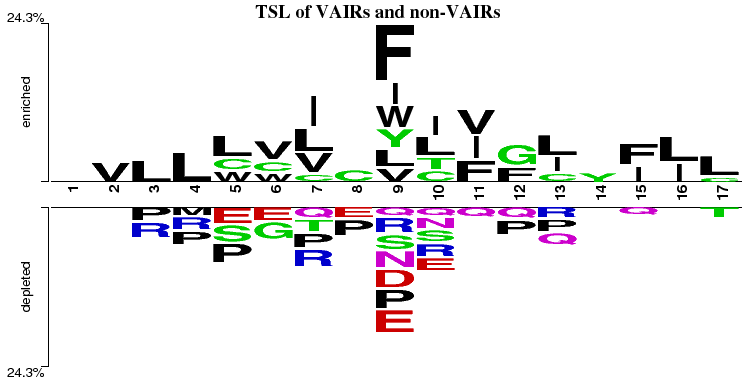


**Figure S7:** The TSL representation of sliding patterns (17-residues length) for prediction of VAIRs. The central residue (9^th^ position) is showing VAIRs (positive) and non-VAIRs (negative).


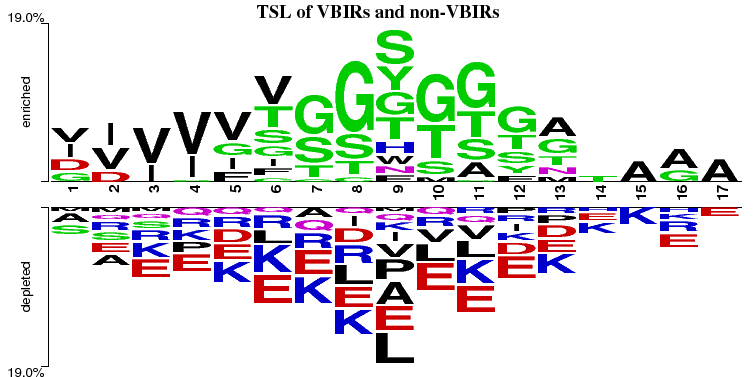


**Figure S8:** The TSL representation of sliding patterns (17-residues length) for prediction of VBIRs. The central residue (9^th^ position) is showing VBIRs (positive) and non-VBIRs (negative).


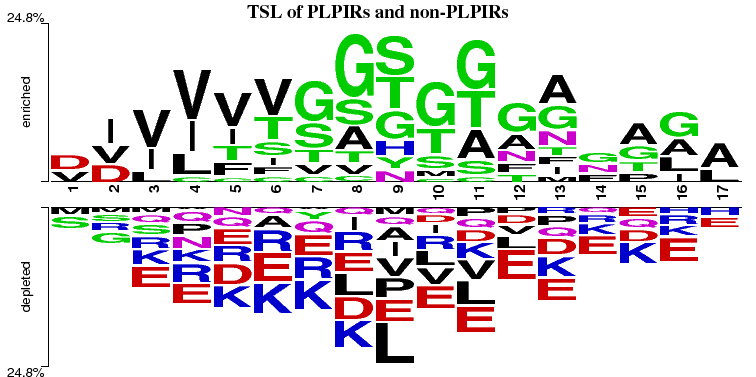


**Figure S9:** The TSL representation of sliding patterns (17-residues length) for prediction of PLPIRs. The central residue (9^th^ position) is showing PLPIRs (positive) and non-PLPIRs (negative).

**Supplementary Table S1:** SVM-based prediction performances (at the default threshold) of binary approach on the different independent datasets.

| **S.No.** | **Prediction** | **Dataset** | **Threshold** | **Sensitivity** | **Specificity** | **Accuracy** | **MCC** |
| --- | --- | --- | --- | --- | --- | --- | --- |
| 1 | VIRs | V-IND-46 | **-0.8** | **62.54** | **68.69** | **68.36** | **0.15** |
|  |  |  | *-0.5* | *24.62* | *95.09* | *91.35* | *0.19* |
| 2 | VAIRs | VA-IND-15 | **-0.8** | **69.61** | **63.78** | **64.43** | **0.21** |
|  |  |  | *-0.1* | *12.15* | *98.96* | *89.27* | *0.23* |
| 3 | VBIRs | VB-IND-27 | **-0.8** | **59.67** | **72.17** | **71.61** | **0.14** |
|  |  |  | *-0.6* | *18.62* | *97.84* | *94.30* | *0.20* |
| 4 | PLPIRs | PLP-IND-16 | **-0.7** | **56.10** | **87.08** | **85.84** | **0.24** |
|  |  |  | *-0.5* | *33.33* | *97.03* | *94.50* | *0.30* |

*Bold value indicates highest performance with balanced sensitivity and specificity.

**Italic value indicates performance with highest MCC.

**Supplementary Table S2:** SVM-based prediction performances of surface accessibility (SA) and Hybrid (PSSM+SA) approaches for four different types of prediction methods on both realistic and balanced datasets. The values of standard errors are also given with performances.

| **Approaches** | **Prediction** | **Realistic datasets** | | | | | **Balanced datasets** | | | | |
| --- | --- | --- | --- | --- | --- | --- | --- | --- | --- | --- | --- |
|  |  | **Thr** | **SN** | **SP** | **ACC** | **MCC** | **Thr** | **SN** | **SP** | **ACC** | **MCC** |
| Surface Accessibility  (SA) | VIRs | **-1.0** | **64.55±1.89** | **46.54±2.15** | **48.15±1.80** | **0.07±0.01** | **0.0** | **55.54±0.73** | **59.62±1.01** | **57.57±0.56** | **0.15±0.01** |
|  |  | *-1.0* | *64.55±1.89* | *46.54±2.15* | *48.15±1.80* | *0.07±0.01* | *0.1* | *50.61±0.94* | *64.86±0.66* | *57.71±0.50* | *0.16±0.01* |
|  | VAIRs | **-1.0** | **71.40±4.43** | **31.16±6.05** | **34.80±5.09** | **0.02±0.01** | **-0.1** | **53.55±2.52** | **52.16±4.00** | **52.83±1.08** | **0.06±0.02** |
|  |  | *-1.0* | *71.40±4.43* | *31.16±6.05* | *34.80±5.09* | *0.02±0.01* | *0.1* | *37.76±3.13* | *69.54±4.68* | *53.66±1.46* | *0.08±0.03* |
|  | VBIRs | **-0.8** | **48.26±0.84** | **67.89±0.43** | **66.13±0.36** | **0.10±0.00** | **-0.1** | **61.54±0.50** | **60.15±1.12** | **60.85±0.75** | **0.22±0.01** |
|  |  | *0.4* | *2.23±0.12* | *99.97±0.01* | *91.21±0.02* | *0.14±0.01* | *0.0* | *56.60±0.57* | *65.92±1.40* | *61.25±0.89* | *0.23±0.02* |
|  | PLPIRs | **-0.9** | **51.19±0.84** | **76.27±1.15** | **74.04±1.02** | **0.18±0.01** | **-0.1** | **66.67±0.54** | **63.40±1.38** | **65.02±0.68** | **0.30±0.01** |
|  |  | *-0.9* | *51.19±0.84* | *76.27±1.15* | *74.04±1.02* | *0.18±0.01* | *-0.1* | *66.67±0.54* | *63.40±1.38* | *65.02±0.68* | *0.30±0.01* |
| Hybrid (PSSM+SA) | VIRs | **-0.8** | **75.96±0.54** | **80.04±0.19** | **79.67±0.20** | **0.37±0.00** | **0.0** | **77.11±0.47** | **76.61±0.47** | **76.86±0.38** | **0.54±0.01** |
|  |  | *0.0* | *46.30±0.71* | *98.84±0.04* | *94.12±0.08* | *0.58±0.01* | *0.1* | *72.84±0.64* | *81.32±0.30* | *77.06±0.27* | *0.55±0.01* |
|  | VAIRs | **-0.9** | **72.68±1.36** | **72.01±0.82** | **72.07±0.66** | **0.28±0.01** | **0.1** | **72.50±1.60** | **71.51±2.35** | **72.01±1.69** | **0.44±0.03** |
|  |  | *-0.1* | *42.57±1.04* | *96.35±0.22* | *91.47±0.21* | *0.43±0.01* | *0.0* | *78.27±2.12* | *65.79±2.30* | *72.01±1.68* | *0.45±0.03* |
|  | VBIRs | **-0.8** | **79.50±0.51** | **83.24±0.15** | **82.90±0.15** | **0.43±0.00** | **0.0** | **82.26±0.73** | **81.75±1.11** | **82.01±0.33** | **0.64±0.01** |
|  |  | *0.1* | *53.52±0.84* | *98.56±0.07* | *94.52±0.06* | *0.62±0.01* | *0.0* | *82.26±0.73* | *81.75±1.11* | *82.01±0.33* | *0.64±0.01* |
|  | PLPIRs | **-0.7** | **90.11±0.75** | **92.31±0.31** | **92.12±0.32** | **0.66±0.01** | **0.0** | **90.20±0.72** | **89.48±0.82** | **89.84±0.49** | **0.80±0.01** |
|  |  | *-0.2* | *79.58±1.05* | *98.67±0.07* | *96.97±0.13* | *0.81±0.01* | *0.0* | *90.20±0.72* | *89.48±0.82* | *89.84±0.49* | *0.80±0.01* |

*Bold value indicates highest performance with balanced sensitivity and specificity.

**Italic value indicates performance with highest MCC.

***If the performance of highest MCC and balanced are at the same threshold, we shown both results separately.

**Supplementary Table S3:** SVM-based prediction performances (at the default threshold) of PSSM approach; according to their total number PSI-BLAST hits of different independent datasets.

| **Prediction** | **Range of total PSI-BLAST Hits** | **Number of sequences** | **Threshold** | **Sensitivity** | **Specificity** | **Accuracy** | **MCC** |
| --- | --- | --- | --- | --- | --- | --- | --- |
| VIRs | Overall (0-500) | 46 | **-0.8** | **73.70** | **71.98** | **72.07** | **0.22** |
|  |  |  | *-0.1* | *41.74* | *96.63* | *93.72* | *0.38* |
|  | 0-10 | 3 | **-0.8** | **59.38** | **65.18** | **64.98** | **0.09** |
|  |  |  | *-0.1* | *3.12* | *96.00* | *92.73* | *-0.01* |
|  | 11-100 | 13 | **-0.8** | **63.83** | **63.99** | **63.98** | **0.15** |
|  |  |  | *-0.1* | *20.21* | *95.94* | *90.62* | *0.19* |
|  | 101-400 | 13 | **-0.8** | **73.33** | **63.83** | **64.44** | **0.19** |
|  |  |  | *-0.1* | *40.56* | *95.52* | *91.97* | *0.35* |
|  | 401-500 | 17 | **-0.8** | **83.07** | **80.24** | **80.36** | **0.31** |
|  |  |  | *-0.1* | *63.39* | *97.54* | *96.09* | *0.56* |
| VAIRs | Overall (0-500) | 15 | **-0.8** | **73.48** | **72.87** | **72.93** | **0.31** |
|  |  |  | *0.0* | *30.39* | *97.22* | *89.77* | *0.37* |
|  | 0-10 | 1 | **-0.8** | **25.00** | **63.64** | **59.46** | **-0.07** |
|  |  |  | *0.0* | *0.00* | *100.00* | *89.19* | *0.00* |
|  | 11-100 | 6 | **-0.8** | **68.92** | **77.13** | **76.04** | **0.34** |
|  |  |  | *0.0* | *12.16* | *97.92* | *86.49* | *0.19* |
|  | 101-400 | 7 | **-0.8** | **74.39** | **67.85** | **68.50** | **0.26** |
|  |  |  | *0.0* | *34.15* | *97.14* | *90.81* | *0.40* |
|  | 401-500 | 1 | **-0.8** | **95.24** | **82.90** | **84.11** | **0.54** |
|  |  |  | *0.0* | *85.71* | *95.34* | *94.39* | *0.73* |
| VBIRs | Overall (0-500) | 27 | **-0.8** | **83.05** | **68.76** | **69.40** | **0.23** |
|  |  |  | *0.1* | *49.40* | *94.49* | *92.47* | *0.35* |
|  | 0-10 | 2 | **-0.8** | **78.57** | **49.11** | **50.06** | **0.10** |
|  |  |  | *0.1* | *21.43* | *90.98* | *88.75* | *0.07* |
|  | 11-100 | 6 | **-0.8** | **82.86** | **55.67** | **57.43** | **0.19** |
|  |  |  | *0.1* | *27.62* | *92.08* | *87.91* | *0.17* |
|  | 101-400 | 6 | **-0.8** | **81.63** | **62.69** | **63.63** | **0.20** |
|  |  |  | *0.1* | *44.90* | *93.50* | *91.08* | *0.30* |
|  | 401-500 | 13 | **-0.8** | **84.57** | **78.90** | **79.12** | **0.29** |
|  |  |  | *0.1* | *68.09* | *96.29* | *95.20* | *0.51* |
| PLPIRs | Overall (0-500) | 16 | **-0.7** | **84.15** | **83.22** | **83.26** | **0.33** |
|  |  |  | *-0.1* | *65.85* | *98.40* | *97.10* | *0.63* |
|  | 0-10 | 2 | **-0.7** | **64.29** | **67.26** | **67.16** | **0.12** |
|  |  |  | *-0.1* | *14.29* | *98.34* | *95.64* | *0.16* |
|  | 11-100 | 0 | - | - | - | - | - |
|  | 101-400 | 3 | **-0.7** | **85.42** | **78.74** | **79.02** | **0.30** |
|  |  |  | *-0.1* | *68.75* | *97.99* | *96.77* | *0.63* |
|  | 401-500 | 11 | **-0.7** | **87.06** | **87.81** | **87.78** | **0.41** |
|  |  |  | *-0.1* | *73.53* | *98.52* | *97.50* | *0.69* |

*Bold value indicates highest performance with balanced sensitivity and specificity.

**Italic value indicates performance with highest MCC.
